# Lysine Catabolism Through the Saccharopine Pathway: Enzymes and Intermediates Involved in Plant Responses to Abiotic and Biotic Stress

**DOI:** 10.3389/fpls.2020.00587

**Published:** 2020-05-21

**Authors:** Paulo Arruda, Pedro Barreto

**Affiliations:** ^1^Centro de Biologia Molecular e Engenharia Genética, Universidade Estadual de Campinas (UNICAMP), Campinas, Brazil; ^2^Departamento de Genética, Evolução e Bioagentes, Instituto de Biologia, Universidade Estadual de Campinas (UNICAMP), Campinas, Brazil; ^3^Genomics for Climate Change Research Center (GCCRC), Universidade Estadual de Campinas (UNICAMP), Campinas, Brazil

**Keywords:** Saccharopine pathway, lysine catabolism, biotic stress, abiotic stress, drought stress

## Abstract

The saccharopine pathway (SACPATH) involves the conversion of lysine into α-aminoadipate by three enzymatic reactions catalyzed by the bifunctional enzyme lysine-ketoglutarate reductase/saccharopine dehydrogenase (LKR/SDH) and the enzyme α-aminoadipate semialdehyde dehydrogenase (AASADH). The LKR domain condenses lysine and α-ketoglutarate into saccharopine, and the SDH domain hydrolyzes saccharopine to form glutamate and α-aminoadipate semialdehyde, the latter of which is oxidized to α-aminoadipate by AASADH. Glutamate can give rise to proline by the action of the enzymes Δ^1^-pyrroline-5-carboxylate synthetase (P5CS) and Δ^1^-pyrroline-5-carboxylate reductase (P5CR), while Δ^1^-piperideine-6-carboxylate the cyclic form of α-aminoadipate semialdehyde can be used by P5CR to produce pipecolate. The production of proline and pipecolate by the SACPATH can help plants face the damage caused by osmotic, drought, and salt stress. AASADH is a versatile enzyme that converts an array of aldehydes into carboxylates, and thus, its induction within the SACPATH would help alleviate the toxic effects of these compounds produced under stressful conditions. Pipecolate is the priming agent of N-hydroxypipecolate (NHP), the effector of systemic acquired resistance (SAR). In this review, lysine catabolism through the SACPATH is discussed in the context of abiotic stress and its potential role in the induction of the biotic stress response.

## Introduction

Plant adaptation to stressful environmental conditions such as drought, salinity, high temperature, and flooding, requires extensive remodeling at the cell, organ, and whole-plant level to sustain structural and metabolic homeostasis. For agriculture, abiotic stress is among the most pressing challenges and, depending on its intensity, frequency, and duration, can cause heavy losses in crop production (Lobell et al., 2011; Ray et al., 2015).

Plants have evolved multiple mechanisms to adapt to biotic and abiotic stress that involve remodeling organelle structure and metabolic pathways associated with protein, nucleic acids, carbohydrates, lipids, amino acids, phytohormones, cations, and small molecule that modulate stress responses (Van Wallendael et al., 2019). Thus, it is expected that not a single component of this integrated network would be capable of modulating cellular and metabolic responses to alleviate the impact of stressful conditions. On the contrary, the performance of plants subjected to biotic and abiotic stress requires a concerted orchestration of the multiple cellular and metabolic remodeling processes. This review focuses on one aspect of amino acid metabolism, lysine catabolism through the saccharopine pathway (SACPATH), which regulates the accumulation of lysine in seeds (Long et al., 2013; and for reviews, see Yu and Tian, 2018; Zhou et al., 2020) and is also involved in abiotic and biotic stress responses (Arruda and Neshich, 2012).

To date, three routes for lysine catabolism have been described in plants: the cadaverine pathway, the SACPATH and the NHP pathway. In the cadaverine pathway, the enzyme lysine decarboxylase converts lysine into the alkaloid cadaverine (Bunsupa et al., 2012). In the SACPATH, that is common to plants, animals, and bacteria, lysine is converted into α-aminoadipate by three enzymatic reactions catalyzed by lysine-ketoglutarate reductase/saccharopine dehydrogenase (LKR/SDH) and α-aminoadipate semialdehyde dehydrogenase (AASADH) (Markovitzs and Chuang, 1987; Papes et al., 1999; Arruda et al., 2000; Azevedo et al., 2003; Struys and Jakobs, 2010; Neshich et al., 2013). In the NHP pathway, lysine is converted into NHP by three reaction steps catalyzed by the enzymes aminotransferase AGD2-like defense response protein (ALD1), which deaminates lysine into dehydropipecolate (DHP), the reductase systemic acquired resistance-deficient 4 (SARD4), which reduces DHP to pipecolate, and flavin-dependent monooxygenase (FMO1), which N-hydroxylates pipecolate to generate NHP (for a review, see Hartmann and Zeier, 2018). NHP is central to plant immunity due to its role in the activation of systemic acquired resistance (SAR) upon pathogen attack (Hartmann and Zeier, 2018). However, the SACPATH has also recently been shown to give rise to pipecolate in a reaction catalyzed by the enzyme Δ^1^-pyrroline-5-carboxylate reductase (P5CR), which is critical for proline biosynthesis (Szabados and Savoure, 2009), on Δ^1^-piperideine-6-carboxylate, the cyclic form of α-aminoadipate semialdehyde (Struys and Jakobs, 2010, Struys et al., 2014). This review is not intended to provide a comprehensive overview of lysine catabolism in plants. Instead, it focuses on the SACPATH peculiarities that gives support to its role in the abiotic stress response and its potential contribution to the biotic stress response. Many excellent reviews concerning biotic stress response are available in the literature and may help complement the ideas and concepts presented in this review.

## Enzymes of the SACPATH

**FIGURE 1:**
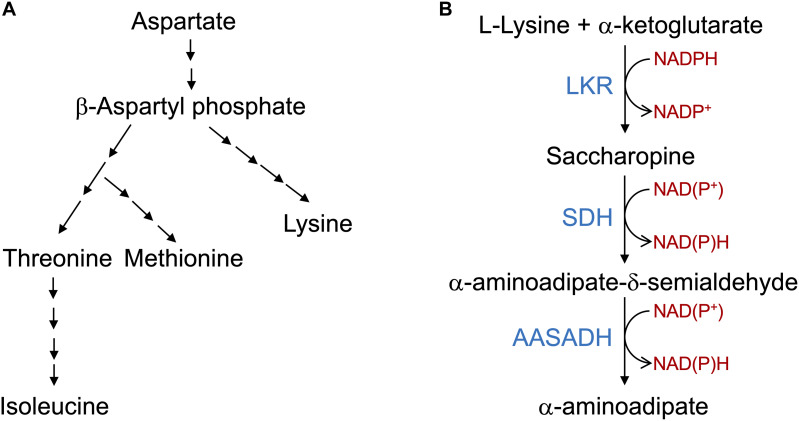
The aspartate pathway for lysine biosynthesis and saccharopine pathway for lysine catabolism. **(A)** Schematic representation of the aspartate pathway. Only the key biosynthetic steps are represented. Aspartate is converted into β-aspartyl phosphate that is used in a serial enzymatic step by steps to produce lysine, threonine, methionine and Isoleucine. The aspartate pathway is localized in the plastids and thus lysine needs to be transported to the cytosol and other organelles. **(B)** The core reactions of the SACPATH involve the conversion of L-lysine into α-aminoadipate. The first two steps of the pathway are catalyzed by the bifunctional enzyme LKR/SDH; the LKR domain condenses L-lysine and α-ketoglutarate into saccharopine using NADPH as a cofactor and the SDH domain hydrolyzes saccharopine into α-aminoadipate semialdehyde and glutamate using NAD(P)^+^ as cofactors. The α-aminoadipate semialdehyde is then oxidized into α-aminoadipate by the enzyme AASADH using NAD(P)^+^ as cofactors.

**FIGURE 2:**
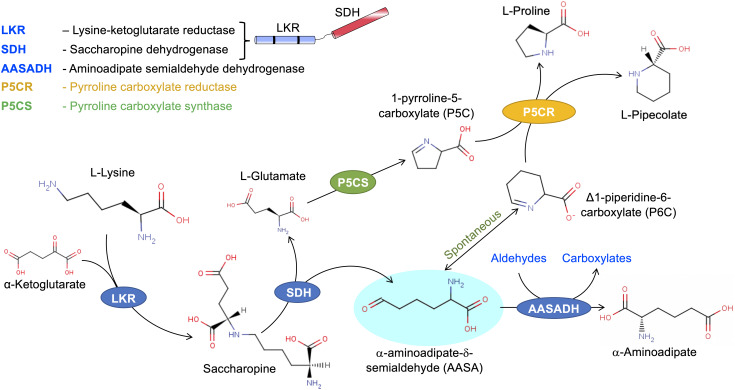
The SACPATH metabolic flux that gives rise to proline and pipecolate. As depicted in Figure 1, the hydrolysis of saccharopine gives rise to glutamate and α-aminoadipate semialdehyde. Glutamate can be converted into proline by the action of P5CS and P5CR. P5CS converts glutamate into Δ^1^-pyrroline 5-carboxylate that is than transformed into proline by P5CR. The α-aminoadipate semialdehyde spontaneously cyclizes to Δ^1^-piperidine 6-carboxylate, which can serve as a substrate for P5CR to produce pipecolate.

The central enzymes of the SACPATH catalyze a transamination-like reaction involving the enzymes LKR/SDH and AASADH (Figure 1B). In this pathway, lysine is condensed with α-ketoglutarate to generate saccharopine using NADPH as a cofactor. Saccharopine is than hydrolyzed to form α-aminoadipate semialdehyde and glutamate using NAD(P)^+^ as a cofactor. Then, AASADH oxidezes α-aminoadioate semialdehyde to α-aminoadioate using NAD(P)^+^ as a cofactor. The glutamate generated in the pathway can be metabolized by Δ^1^-pyrroline-5-carboxylate synthetase (P5CS) and P5CR to produce proline while the cyclic form of α-aminoadipate semialdehyde, Δ^1^-piperideine-6-carboxylate, can be used by P5CR to produce pipecolate (Figure 2). The catalytic properties and subcellular localization of each of these enzymes may help in the understanding of their metabolic role in stress response.

### LKR/SDH

The enzyme LKR/SDH was first demonstrated to occur in plants in developing maize endosperm (Arruda et al., 1982; Brochetto-Braga et al., 1992). LKR and SDH belong to a single ∼120 kDa bifunctional polypeptide (Gonçalves-Butruille et al., 1996; Gaziola et al., 1997) that localizes in the cytosol (Kemper et al., 1999) in contrasts with the animal LKR/SDH, that localizes in the mitochondria matrix (Markovitzs and Chuang, 1987). Immature endosperms of high-lysine maize mutants, in addition to the bifunctional LKR/SDH polypeptide, also presents a small proportion of an active monofunctional SDH (Pompeu et al., 2006). Monofunctional SDH have also been found in animals (Papes et al., 1999) and in Arabidopsis (Stepansky et al., 2005). A monofunctional SDH have also been detected in Arabidopsis mitochondria (Fuchs et al., 2019). In animals, SDH activity seems to be essential inside mitochondria, as the organelle, in addition to the bifunctional LKR/SDH polypeptide, have significant amounts of a monofunctional SDH (Zhou et al., 2018).

In immature maize and rice endosperm, the enzymatic activity of the LKR domain is activated by Ca^2+^, Mg^2+^, high salt, and osmolytes (Kemper et al., 1998; Gaziola et al., 2000). The LKR domain, when separated from the SDH domain by limited proteolysis, retains its Ca^2+^ activation. However, it is inhibited by a protein fraction containing SDH polypeptides, suggesting that the SDH domain appears to negatively regulate the activity of the LKR domain (Kemper et al., 1998). The LKR domain but not the SDH domain is also activated by phosphorylation in a lysine-dependent manner (Arruda et al., 2000; Azevedo and Lea, 2001). In plants, the LKR and SDH domains of the bifunctional polypeptide are separated from each other by an ∼130 amino acid interdomain (Kemper et al., 1999; Anderson et al., 2010). Genome sequencing of genes encoding LKR/SDH revealed that, in most plants, the enzyme is encoded by a single gene, with a lone exception reported for poplar, which has two functional genes. Interestingly, transcripts encoding monofunctional LKR and monofunctional SDH have been identified in several plant species (Anderson et al., 2010).

### AASADH

The AASADH enzyme belongs to the superfamily of NAD(P)^+^-dependent aldehyde dehydrogenases (ALDs) that catalyze the conversion of aliphatic and aromatic aldehydes into their corresponding carboxylates (Singh et al., 2012). AASADH can use an array of aldehydes as substrates (Brocker et al., 2010; Kyiota et al., 2015) but seems to be the only ALD capable of using α-aminoadipate semialdehyde as a substrate, as null mutations of this enzyme in humans cause the disease pyridoxine-dependent epilepsy (PDE) (Mills et al., 2006). The ability of AASADH to use multiple aldehydes as substrates, such as betaine aldehyde, hexanal, octanal, non-anal, trans-2-non-anal, benzaldehyde and propanal (Brocker et al., 2010; Kyiota et al., 2015), makes it a scavenger of aldehydes generated by lipid peroxidation, carbohydrate metabolism, amino acid catabolism, and oxidative stress, among others. AASADH has gained attention for its roles in the abiotic stress response and its involvement in human PDE (Mills et al., 2006; Rodrigues et al., 2006; Brocker et al., 2010, 2011; Kyiota et al., 2015; Crowther et al., 2019; Yang et al., 2019).

A detailed characterization of an AASADH enzyme was performed in a study of its role in hyperosmotic protection and cellular homeostasis in mouse (Brocker et al., 2010). AASADH is encoded by a single gene that generates differentially spliced transcripts producing polypeptides targeted to the cytosol and to the mitochondria. The transcript encoding the mitochondrial-targeted AASADH uses an alternative start codon that adds a mitochondrial signal sequence to the amino-terminus of the protein (Brocker et al., 2010).

Interestingly, compared with that for α-aminoadipate semialdehyde, the Km for AASADH from both animals and maize was shown to be 4-20-fold lower for non-anal, hexanal, octanal, benzaldehyde, and propanal (Brocker et al., 2010; Kyiota et al., 2015). The much higher affinity for the aliphatic aldehydes suggests an essential role of AASADH in alleviating the cellular toxicity of aldehydes, especially when plants are subjected to stress.

### P5CS

P5CS is a bifunctional polypeptide containing γ-glutamyl kinase (GK) and glutamate-5-semialdehyde dehydrogenase (GPR) domains (Zhang et al., 1995; Perez-Arellano et al., 2010; Verslues and Sharma, 2010). In plants, there are two isoforms, P5CS1 and P5CS2, which are encoded by two closely related genes (Szekely et al., 2008). P5CS1 localizes in chloroplasts, whereas P5CS2 localizes to the cytosol. The P5CS amino acid sequence is highly conserved across species, which suggests its general biological relevance. The GPR catalytic domain converts γ-glutamyl phosphate to glutamate-5-semialdehyde, which then spontaneously cyclizes into Δ^1^-pyrroline-5-carboxylate (Verslues and Sharma, 2010) (Figure 2). P5CS is a rate-limiting enzyme and thus is critical in increasing proline concentrations when plants are subjected to abiotic stress (Szabados and Savoure, 2009; Verslues and Sharma, 2010; Bhaskara et al., 2015). GK activity, but not GPR activity, is subject to feedback inhibition by proline, implying that the level of free amino acids in cellular medium feedback regulates enzyme activity (Zhang et al., 1995).

### P5CR

P5CR has been less studied in plants. The enzyme converts Δ^1^-pyrroline-5-carboxylate into proline (Figure 2); it is ubiquitous to most plant organs, and its expression is particularly high in soybean root nodules (Szoke et al., 1992). The enzyme is encoded by a single gene and has a 29 kDa monomeric native form. P5CR is mostly localized in the cytosol, although a small proportion of the enzyme also localizes in plastids (Szoke et al., 1992). Despite being encoded by a single gene, two isoforms of the enzymes P5CR-1 and P5CR-2 have been identified in spinach: P5CR-2 is a plastid isoform (Verslues and Sharma, 2010). P5CR is not rate limiting for the synthesis of proline and its expression seems not to be induced by abiotic stress (Verslues and Sharma, 2010), although upregulation of expression of the enzyme has been shown in plants and bacteria subjected to osmotic/salt stress (Neshich et al., 2013; Koenigshofer and Loeppert, 2019).

## Metabolic Flux Through the SACPATH

In higher eukaryotes, the metabolic flux from lysine to α-aminoadipate (Figure 2) is unidirectional, since there is no evidence of the involvement of the SACPATH in lysine biosynthesis. In certain bacteria, lysine is directly oxidized into α-aminoadipate semialdehyde by the enzyme lysine dehydrogenase (Neshich et al., 2013). However, α-aminoadipate semialdehyde is highly toxic to eukaryotic cells. In humans, for example, its accumulation causes PDE (Mills et al., 2006). Thus, α-aminoadipate semialdehyde must be maintained at low concentrations by AASADH and P5CR to produce α-aminoadipate and pipecolate, respectively (Figure 2). Metabolic flux toward α-aminoadipate and pipecolate occurs when plants are exogenously fed lysine (Moulin et al., 2006; Kyiota et al., 2015). The glutamate formed by saccharopine hydrolysis can be used for the synthesis of proline (Figure 2). Proline synthesis from lysine was first shown by C^14^-lysine feeding in the immature endosperm of wheat, maize, and barley (Lawrence and Grant, 1964; Sodek and Wilson, 1970; Brandt, 1975). Although LKR/SDH enzymes were unknown at that time, the SACPATH must have been responsible for the conversion of lysine to proline.

The conversion of Δ^1^-piperideine-6-carboxylate to pipecolate (Figure 2) was demonstrated in animals in a study designed to investigate whether fibroblasts catabolize lysine through the SACPATH or through the pipecolate pathway, both of which converges to α-aminoadipate semialdehyde (Struys and Jakobs, 2010). A human AASADH-deficient fibroblast cell line fed either L-[α-^15^N] or L-[ε-^15^N]lysine catabolized L-[α-^15^N]lysine into [α-^15^N]saccharopine, [α-^15^N]aminoadipic semialdehyde, [α-^15^N]Δ^1^-pyrroline-6-carboxylate, and [α-^15^N]pipecolate (Struys and Jakobs, 2010). The incubation of recombinant human P5CR with Δ^1^-piperideine-6-carboxylate further demonstrated its enzymatic conversion solely into pipecolate (Struys et al., 2014). Previously, studies in an animal model argued that the pipecolate pathway is preferred for lysine catabolism in the brain, while the SACPATH is preferred for the liver and kidney (Sauer et al., 2011; Hallen et al., 2013; Posset et al., 2014). An accurate experimental design to follow the time course of lysine degradation in the mouse liver, kidney, and brain revealed that the catabolic activity peak in response to exogenous lysine fed to the animals occurs 2 h after intraperitoneal injection of lysine (Pena et al., 2016). By the use of this experimental setup, intraperitoneal injection of L-[α-^15^N] or L-[ε-^15^N]lysine revealed that lysine catabolism in the mouse liver, kidney, and brain occurs through the SACPATH (Pena et al., 2017). There was no evidence for the synthesis of α-aminoadipate from the pipecolate pathway. In addition, pipecolate derived from L-[α-^15^N]lysine confirmed its synthesis through the SACPATH in the liver, kidney, and brain (Pena et al., 2017). The prevalence of the SACPATH for lysine catabolism and pipecolate synthesis was also recently observed for human neuronal progenitor cells and fibroblasts. Isotopic tracing experiments, similar to those carried out in mice, revealed that lysine catabolism occurs exclusively through the SACPATH (Crowther et al., 2019). L-[α-^15^N] or L-[ε-^15^N]lysine feeding experiments in bean also indicate preferred incorporation of the α-N group into pipecolate (Schutte and Seeling, 1967 was coted in the text as cited by Hartmann et al., 2017).

## Involvement of the SACPATH in the Abiotic and Biotic Stress Response

Lysine catabolism through the SACPATH is highly responsive to abiotic stress (Moulin et al., 2006; Boex-Fontvieille et al., 2013; Kyiota et al., 2015; Michaletti et al., 2018; Wu et al., 2018; Dudziak et al., 2019; Yadav et al., 2019; You et al., 2019). Nevertheless, to date, there is no direct evidence for the involvement of the SACPATH in the biotic stress response. Instead, the recent discovery of the NHP pathway of lysine catabolism has shown its critical role for the induction of SAR (for a review, see Hartmann and Zeier, 2018).

In the previous sections, we presented the current knowledge of the enzymatic properties that support the synthesis through the SACPATH of two of the most critical osmolytes, proline and pipecolate (Szabados and Savoure, 2009; Bhaskara et al., 2015; Pérez-García et al., 2019). However, the question is how the SACPATH enzymatic activities and derived metabolites contribute to abiotic and biotic stress responses. The response of the SACPATH to osmotic, drought and salt stress is conserved in plants, animals, and bacteria, indicating its evolutionary importance to cope with abiotic stress. Notably, the enzymes LKR/SDH and AASADH are co-upregulated at the transcriptional level by exogenously applied lysine in plants, animals, and bacteria (Papes et al., 1999; Stepansky et al., 2005; Neshich et al., 2013; Kyiota et al., 2015). The induction of LKR activity by phosphorylation in a lysine-dependent manner implies that this enzyme is quickly activated to produce saccharopine once lysine levels start rising. The immediate increase in LKR activity stimulates increases in SDH activity, as the two activities occur within the same polypeptide. The immediate consequence of these two reaction steps is the increase in the concentration of α-aminoadipate semialdehyde, which would require an increase in AASADH and perhaps P5CR activities to maintain α-aminoadipate semialdehyde concentrations below toxic levels.

Metabolic perturbations such as those imposed by osmotic, drought and salt stress, will lead to the recycling of cellular catabolic processes, including proteomic hydrolysis (for a review, see Hildebrandt et al., 2015). Stress-induced protein hydrolysis results in increased free lysine levels. Increased lysine pool could also result from the induction of the aspartate (AK) pathway for lysine biosynthesis (Figure 1A) that has also been shown to be induced under abiotic stress (Pratelli and Pilot, 2014). However, the AK pathway for lysine biosynthesis localizes in the chloroplast and the amino acid needs to be transported to the cytosol and other organelles to be effectively metabolized. One of the most well-characterized amino acid transporters that operate in the chloroplast is the LHT1, which transport lysine and histidine (Fischer et al., 1998; Pratelli and Pilot, 2014). LHT1 has also been shown to be induced under abiotic stress and thus connect lysine biosynthesis to its catabolism in an integrated stress response metabolic network (Pratelli and Pilot, 2014). Increased free lysine levels have been observed in plants subjected to abiotic stress, but of relevance is the observation of higher levels of free lysine in sesame and wheat drought-tolerant genotypes compared with susceptible genotypes subjected to drought stress (Yadav et al., 2019; You et al., 2019). These observations suggest that abiotic stress-tolerant genotypes may have selected traits associated with the upregulation of the SACPATH. Indeed, transcriptomic, proteomic and metabolomic profiling of abiotic stress-tolerant sesame and wheat genotypes have revealed an induction of metabolic flux throughout the SACPATH to a greater extent in drought-tolerant genotypes than in drought-susceptible genotypes, which resulted in the accumulation of saccharopine and α-aminoadipate (Michaletti et al., 2018; You et al., 2019).

The role of the SACPATH in the osmotic stress response was first reported in rapeseed leaf disks treated with polyethylene glycol solutions at −1 to −4 MPa (Moulin et al., 2006). It was found that hyperosmotic treatment of rapeseed leaf disks induces an increase in LKR/SDH transcript abundance and enzymatic activity, which correlates with decreased levels of free lysine and increased levels of pipecolate (Moulin et al., 2006). Furthermore, the LKR/SDH activity and pipecolate concentration decrease with the return of the leaf disks to hypoosmotic conditions (Moulin et al., 2006). Exogenous administration of lysine induces LKR/SDH activity and increases pipecolate levels to the same extent as does hyperosmotic treatment, which supports the hypothesis that pipecolate is synthesized via the SACPATH (Moulin et al., 2006). Other abiotic stresses, such as those caused by different illumination conditions, also induce the SACPATH. A metabolic genome-wide association study of 309 Arabidopsis accessions subjected to light and dark treatments revealed a strong stress association between saccharopine accumulation and single-nucleotide polymorphisms (SNPs) at LKR/SDH loci. Saccharopine accumulates under low-light or dark conditions, indicating that the induction of the SACPATH is essential under these stressful conditions (Wu et al., 2018). Moreover, the osmolytes proline and pipecolate accumulate to high levels in halophytes, indicating their role in adaptative metabolism under stressful saline conditions (Slama et al., 2015; Jawahar et al., 2019).

The lysine-to-α-aminoadipate metabolic flux requires the coordinated activities of LKR/SDH and AASADH. Indeed, LKR/SDH, and AASADH are co-upregulated at transcriptional and translational levels in response to exogenous administration of lysine in plants, animals, and bacteria (Neshich et al., 2013; Kyiota et al., 2015; Pena et al., 2016, 2017). The synthesis of pipecolate from Δ^1^-piperideine-6-carboxylate by the enzyme P5CR (Struys and Jakobs, 2010; Struys et al., 2014; Pena et al., 2017) suggest that the SACPATH contributes to abiotic stress alleviation by increasing cellular concentrations of both proline and pipecolate (Figure 2). However, the accumulation of saccharopine when plants are subjected to abiotic stress (Michaletti et al., 2018; You et al., 2019) must be further investigated. In animals, the SACPATH is exclusively located in the mitochondria (Blemings et al., 1994), an organelle essential for sustaining cellular energy homeostasis under stress conditions. However, the accumulation of saccharopine is toxic to mitochondria (Zhou et al., 2018). In humans, mutations in the LKR domain of the LKR/SDH enzyme lead to lysinuria, an asymptomatic disease (Sacksteder et al., 2000), but mutations in the SDH domain lead to saccharopinuria, a disease associated with severe brain malfunction and retardation (Houten et al., 2013). Saccharopinuria leads to the accumulation of high levels of saccharopine, which induces mitochondrial malfunction that can be reversed by the expression of a wild-type mitochondrial-targeted SDH (Zhou et al., 2018). In Arabidopsis, a monofunctional SDH probably produced from the same gene encoding the bifunctional enzyme localizes in the mitochondria (Fuchs et al., 2019). Under stress conditions, the targeting of SDH to the mitochondria may help reduce the concentration of saccharopine inside the organelle and maintain its normal function. Another point of significance of the SACPATH when plants are subjected to abiotic stress is the synthesis α-aminoadipate semialdehyde. α-Aminoadipate semialdehyde should be maintained at a low level because it is highly reactive; can bind to proteins, nucleic acids, and small molecules; and can interfere with an array of cellular processes. It is possible that α-aminoadipate semialdehyde acts in the upregulation of AASADH (Brocker et al., 2010, 2011; Kyiota et al., 2015) to help rid cells of aldehydes generated from stress-induced metabolic processes (Singh et al., 2012). Additionally, AASADH can produce other osmolytes such as betaine from betaine aldehyde that would help alleviate stress imposed by salt, osmotic, and drought (Singh et al., 2012) and also sustain cellular energy homeostasis (Yang et al., 2019).

**FIGURE 3:**
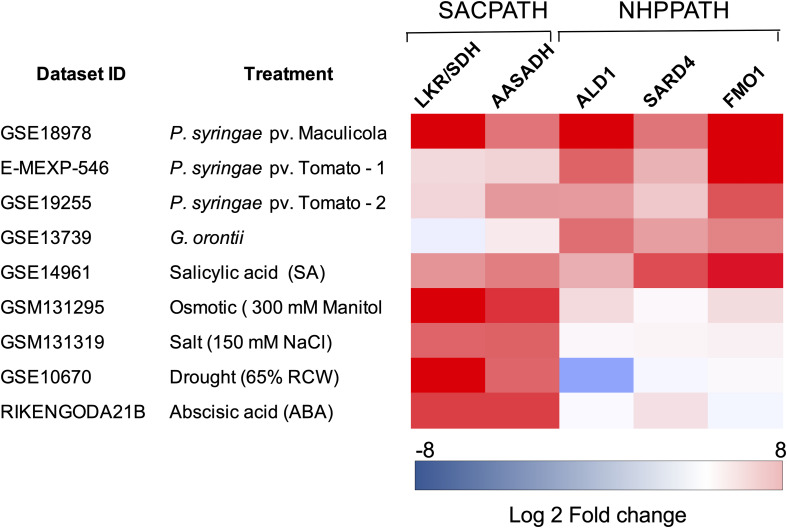
Expression profiling of the core genes of the SACPATH and NHP pathway for lysine catabolism in Arabidopsis subjected to biotic and abiotic stresses. Expression profiling datasets of the genes encoding LKR/SDH and AASADH of the SACPATH and ALD1, SARD4, and FMO1 of the NHP pathway, were retrieved from public databases and analyzed using the Genevestigator platform^1^. Only the data of wild type (Col-0 or Ws-0) subjected to control and stress treatments were used in this analysis. The following datasets were used: **GSE18978**, leaves inoculated or not inoculated with *Pseudomonas syringae* ES4326; **E-MEXP-546**, leaves inoculated or not inoculated with the *P. syringae* pv. tomato avirulent strain DC3000 avrRpm1; **GSE19255**; leaves inoculated or not inoculated with *P, syringae* pv. tomato; **GSE13739**, leaves inoculated or not inoculated with the *G. orontii* MGH isolate; **GSE14961**, seedlings grown for 1 day in 2 mM salicylic acid; **GSM131295**, 16-day-old seedlings grown in 300 mM mannitol (osmotic stress); **GSM131319**, 16-day-old seedlings grown in 150 mM NaCl (salt stress); **GSE10670**, plants subjected to drought stress; and **RIKENGODA21B**, seedlings treated with 10 μM ABA. The values are expressed as log2 of the fold change between treated and untreated plants.

Another relevant question is whether the pipecolate generated by the SACPATH contributes to the stimulation of the plant immune response in addition to pipecolate produced through the NHP pathway (Hartmann et al., 2017, 2018; Chena et al., 2018; Hartmann and Zeier, 2018; Wang C. et al., 2018; Wang Y. et al., 2018). Pipecolate accumulates to high levels upon pathogen attack, stimulating the synthesis of NHP, which subsequently activates SAR at the infected site and in distant leaves (Hartmann and Zeier, 2018). Engineering the NHP pathway by the transient overexpression of ALD1 and FMO1 has been shown to activate the SAR response in tomato plants (Holmes et al., 2019). Is the pipecolate produced by the NHP pathway the only pipecolate source for SAR activation or can the pipecolate produced by the SACPATH contribute, at least in part, to immune response? Perhaps the different intracellular localization of the SACPATH and the NHP pathway may have a role in differentiating the pipecolate source in the activation of the biotic and abiotic stress response. The bifunctional LKR/SDH, at least in immature maize endosperm, is located in the cytosol, but a monofunctional SDH has been found that targets the mitochondria. AASADH is localized both in the cytosol and in the mitochondria but not in the plastids. The intracellular localization of P5CR is not well established, but it has also been found in plastids. The NHP pathway seems to be localized in the chloroplasts of developed leaves. Thus, it is possible that the pipecolate produced from the SACPATH and NHP is differentially compartmentalized so that the NHP pathway, whose enzymes are located in the chloroplast, is directly associated with the biotic stress response and therefore is preferentially associated with pathogen infection. We took advantage of public datasets designed to study the transcriptomic profiling of Arabidopsis to interrogate whether the SACPATH and NHP pathway are differentially expressed under biotic and abiotic stress. A heatmap was constructed to profile the transcriptional response of LKR/SDH, AASADH, ALD1, SARD4, and FMO1 in Arabidopsis subjected to biotic and abiotic stress conditions (Figure 3). Although the datasets were retrieved from distinct experiments, we can clearly observe that the expression of the genes encoding the enzymes of the SACPATH and the NHP pathway are co-upregulated under biotic stress imposed by infection with *P. syringae* or treatment with salicylic acid (Figure 3). In the case of infection with the fungus *Golovinomyces orontii*, only the NHP pathway is upregulated. In contrast, under abiotic stress imposed by osmotic, salt, or drought stress or when plants were treated with abscisic acid (ABA), the SACPATH was preferentially upregulated (Figure 3). This means that the SACPATH may contribute to the synthesis of NHP from the pipecolate produced from Δ^1^-pyrroline-6-carboxylate through the enzymatic activity of P5CR (Figure 3) but the NHP pathway may not contribute with abiotic stress response.

## Conclusion

**FIGURE 4:**
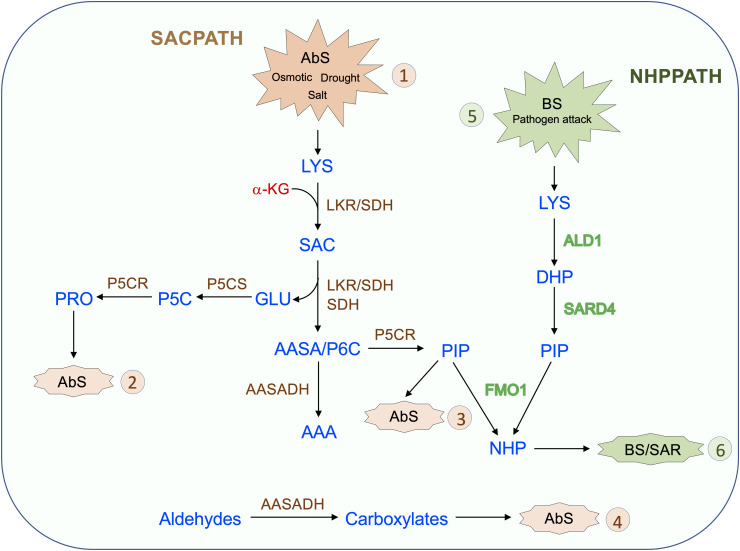
Proposed model for the role of the SACPATH in abiotic and biotic stress responses in plants. Upon abiotic stress (AbS, 1) such as that imposed by osmotic, salt, and drought, the cellular concentration of free lysine increase. As the lysine concentration increases, the transcripts encoding bifunctional LKR/SDH, monofunctional SDH, and AASADH accumulate, increasing the lysine-to-α-aminoadipate metabolic flux. In general, the expression of P5CS and P5CR is also upregulated under abiotic stress, and by the action of these two enzymes, glutamate is converted into proline (AbS, 2), and Δ^1^-piperidine 6-carboxylate is converted into pipecolate (AbS, 3). Proline and pipecolate are the two most effective osmolytes and help sustain cellular homeostasis under abiotic stress. The upregulation of AASADH helps metabolize aldehydes generated by stress conditions, including, for example, betaine aldehyde, whose conversion generates betaine, another important osmolyte (AbS, 4). Upon pathogen attack (BS, 5) lysine is also catabolized to pipecolate through the NHP pathway by the enzymes ALD1, which deaminates lysine to Dhp, and SARD4, which reduces Dhp to pipecolate. Dhp is then N-hydroxylated by FMO1 to generate NHP, the effector of SAR (BS, 6). In the proposed model, the pipecolate generated by the SACPATH could also serve as a substrate for FMO1 to produce NHP, therefore contributing to SAR.

The SACPATH for lysine catabolism is widespread across kingdoms, except for fungi, which use the pathway for lysine synthesis. Upon intracellular increase in lysine concentration, due to metabolism-related organ development programs or responses to abiotic and biotic stress, LKR undergoes posttranscriptional modification mediated by Ca^2+^ and phosphorylation to initiate SACPATH reactions, first by LKR activity to produce saccharopine and then by SDH activity to produce α-aminoadipate semialdehyde and glutamate (Figure 4, Abs 1). Increased intracellular free lysine also induces the transcriptional upregulation of the *LKR/SDH* and *AASADH* genes which boost the lysine-to-α-aminoadipate metabolic flux and the use of glutamate to produce proline (Figure 4, Abs 2) and the intermediary D^1^-piperideine-6-carboxylate to produce pipecolate (Figure 4, Abs 3). A monofunctional SDH enzyme is targeted to the mitochondria, perhaps to help maintain saccharopine levels below toxic levels. The upregulation of AASADH in turn would help to maintain α-aminoadipate semialdehyde at low levels and help to reduce the levels of the stress-generated aldehydes, thus contributing to the alleviation of the toxic effects on cellular metabolism (Figure 4, Abs 4). The proline and pipecolate generated by the SACPATH must contribute to alleviating the cellular impact of osmotic, salt, and drought stress (Figure 4, Abs 1). However, the cellular availability of pipecolate generated by the SACPATH could also contribute to the production of NHP and thus induce SAR, as the enzymes LKR/SDH and AASADH are induced by pathogen attack (Figure 3). Perhaps the main difference between the biotic and abiotic stress responses associated with lysine catabolism through the SACPATH and the NHP pathway resides in the mode of action of these two conditions. Pathogen infection is punctual (Figure 4, BS 5), and, independent of the plant organ, the infection spots comprise a limited number of cells. At the infection site, lysine catabolism through the NHP pathway must be intense, pipecolate accumulates at high levels, and NHP is produced and spread over long distances to mediate the SAR response (Figure 4, BS 6). However, a prerequisite to start the process is the rise of free lysine concentration in the infection site. The rise of free lysine concentrations independent, whether it is due to biotic or abiotic stress, induces the SACPATH (Figure 3) that can also mediate the synthesis of NHP. In contrast, osmotic, drought, and salt stress affect whole plant organs simultaneously, leading to the activation of the SACPATH and accumulation of pipecolate. Since abiotic stress must debilitate the plant, making it more prone to pathogen attack, the pipecolate produced by the SACPATH may help to stimulate the immune response in these circumstances.

## Author Contributions

PA organized and wrote the manuscript. PB contributed with the expression profiling analysis of SACPATH and NHP pathways.

## Conflict of Interest

The authors declare that the research was conducted in the absence of any commercial or financial relationships that could be construed as a potential conflict of interest.

## Footnotes

1 https://genevestigator.com/gv/
